# Selective isotope labeling for NMR structure determination of proteins in complex with unlabeled ligands

**DOI:** 10.1007/s10858-019-00241-9

**Published:** 2019-04-30

**Authors:** Konstantinos Tripsianes, Ulrike Schütz, Leonidas Emmanouilidis, Gerd Gemmecker, Michael Sattler

**Affiliations:** 1 CEITEC—Central European Institute of Technology, Masaryk University, Kamenice 5, 62500 Brno, Czech Republic; 2 Institute of Structural Biology, Helmholtz Zentrum München, Ingolstädter Landstr. 1, 85764 Neuherberg, Germany; 3 Center for Integrated Protein Science Munich at Chair of Biomolecular NMR, Department Chemie, Technische Universität München, Lichtenbergstr. 4, 85747 Garching, Germany

**Keywords:** NMR spectroscopy, Isotope labeling, Protein–ligand interactions, NOE

## Abstract

The physiological role of proteins is frequently linked to interactions with non-protein ligands or posttranslational modifications. Structural characterization of these complexes or modified proteins by NMR may be difficult as the ligands are usually not available in an isotope-labeled form and NMR spectra may suffer from signal overlap. Here, we present an optimized approach that uses specific NMR isotope-labeling schemes for overcoming both hurdles. This approach enabled the high-resolution structure determination of the farnesylated C-terminal domain of the peroxisomal protein PEX19. The approach combines specific ^13^C, ^15^N and ^2^H isotope labeling with tailored NMR experiments to (i) unambiguously identify the NMR frequencies and the stereochemistry of the unlabeled 15-carbon isoprenoid, (ii) resolve the NMR signals of protein methyl groups that contact the farnesyl moiety and (iii) enable the unambiguous assignment of a large number of protein-farnesyl NOEs. Protein deuteration was combined with selective isotope-labeling and protonation of amino acids and methyl groups to resolve ambiguities for key residues that contact the farnesyl group. Sidechain-labeling of leucines, isoleucines, methionines, and phenylalanines, reduced spectral overlap, facilitated assignments and yielded high quality NOE correlations to the unlabeled farnesyl. This approach was crucial to enable the first NMR structure of a farnesylated protein. The approach is readily applicable for NMR structural analysis of a wide range of protein–ligand complexes, where isotope-labeling of ligands is not well feasible.

**Electronic supplementary material:**

The online version of this article (10.1007/s10858-019-00241-9) contains supplementary material, which is available to authorized users.

## Introduction

Detailed understanding of the mechanism for protein–ligand interactions or the effects of posttranslational modifications requires structural information about the ligand-bound protein of interest on a molecular level. These complexes or modified proteins often possess intrinsic flexibility or low ligand affinity and are difficult to crystallize in the native conformation. Structural analysis by NMR on the other hand, can be problematic as isotope-labeling of the ligands may not be readily available, thus narrowing down the options to derive NOE-based distance restraints to ^1^H only homonuclear experiments. However, homonuclear experiments suffer from spectral overlap, and thus render investigations of more complex systems involving larger proteins or chemically complex ligands challenging or unfeasible. On the other hand, various approaches for selective isotope enrichment of proteins based on metabolic percursors have been developed in the past decade and are mostly commercially available (Tugarinov et al. 2006; Kerfah et al. 2015).

Protein prenylation, a class of lipid modification involving covalent addition of either farnesyl or geranylgeranyl groups, plays diverse roles in the regulation of trafficking, signaling and behavior of cellular proteins (Miura and Treisman 2006; Sorek et al. 2009; Novelli and D’Apice 2012). It is estimated that over 100 human proteins are potential substrates for farnesylation and geranylgeranylation (McTaggart 2006). Many of these proteins belong to the superfamily of small guanine nucleotide triphosphatases (GTPases) (Takai et al. 2001).

The peroxisomal membrane protein receptor PEX19 is farnesylated in vivo and the lipid modification is necessary for its critical function in peroxisomal biogenesis (Emmanouilidis et al. 2017; Rucktäschel et al. 2009). The 15-carbon isoprenoid, donated by farnesyl diphosphate (FPP), is transferred by protein farnesyl transferases to a conserved cysteine at the C-terminal CaaX motif (C = cysteine, aa = two aliphatic amino acids, X = any amino acid) (Manne et al. 1990). While the crystal structure of a C-terminal fragment of the PEX19 domain has been solved (Schueller et al. 2010), crystallization attempts with the farnesylated protein have failed. Therefore, solution state NMR techniques were applied for structural investigations of the farnesylated PEX19 C-terminal domain (residues 161–299), herein referred to as PEX19.

**Fig. 1:**
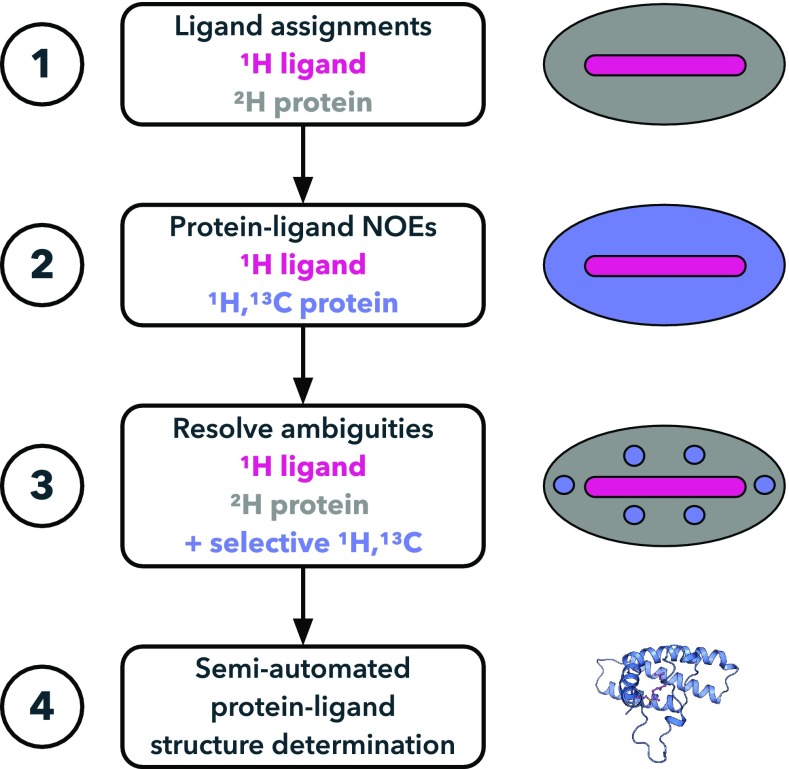
Flowchart and NMR labeling schemes that allow identifying ligand frequencies (1), collecting protein–ligand NOEs (2), and resolving NOE ambiguities (3) for structure determination of protein–ligand complexes (4)

In this study, we made use of optimized isotope labeling of precursors in different protein labeling schemes. The combination of the various labeling protocols with individually adapted NMR experiments enabled us to circumvent the restrictions for the unlabeled ligand, to obtain complete resonance assignments for both protein and the attached lipid and to determine the first NMR structure of a farnesylated protein (Emmanouilidis et al. 2017) (Fig. 1).

## Results

### NMR analysis of the farnesyl resonances

**Fig. 2:**
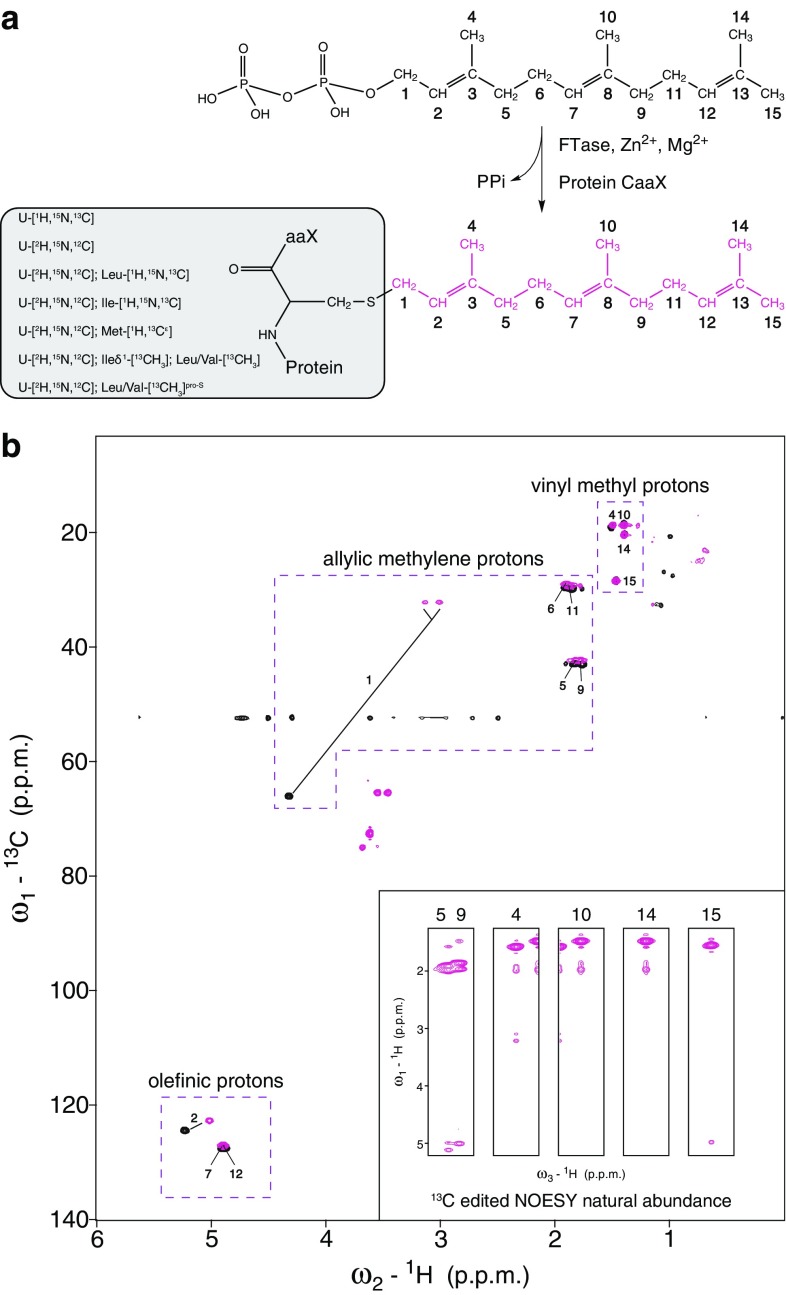
Sampling the farnesyl resonances. **a** Schematic representation of in vitro farnesylation reaction for any target protein containing the CaaX box. Farnesyl groups are numbered. Any labeling scheme, as the ones listed for the present study, can be applied to the protein before the attachment of the unlabeled farnesyl. **b** Natural abundance ^13^C,^1^H HSQC spectra of farnesylated PEX19 labeled with [^2^H]-glucose (magenta) and farnesyl pyrophosphate dissolved in methanol (black). In either case, farnesyl resonances are numbered as shown in **a**. Inset shows NOE strips from a natural abundance ^13^C-edited 3D NOESY spectrum recorded on perdeuterated PEX19 using non-uniform sampling. Numbers at the top indicate the edited farnesyl resonances

The farnesyl moiety is a linear grouping of three isoprene units that is attached enzymatically to the cysteine of the C-terminal CaaX box of PEX19. The reaction can be performed in vitro using purified PEX19, the farnesyl transferase enzyme, farnesyl pyrophosphate as the donor of (2*E*,6*E*)-farnesol, and cofactors such as Mg^2+^ and Zn^2+^ to obtain fully farnesylated PEX19 protein (Emmanouilidis et al. 2017) (Fig. 2a). All protein samples described herein refer to farnesylated PEX19. PEX19 labeling can vary but the farnesyl is always at natural isotopic abundance.

For characterizing the farnesyl group, we first used a PEX19 sample labeled uniformly with ^15^N and ^13^C, on which all the standard NMR experiments were performed. We applied isotope filters and recorded homonuclear NOESY and TOCSY spectra in D_2_O (Breeze 2000; Sattler et al. 1999). By comparing the proton correlations in both spectra we could identify one isoprenoid group due to the very characteristic resonances of a pair of allylic methylene protons (Fig. S1). Inspection of the NOE correlations originating from the methylene protons of the lipidated cysteine in a 3D ^13^C-edited NOESY spectrum (with or without isotope filters in ω_1_) indicated that this is the first isoprenoid unit of the farnesyl (Fig. S1). Further farnesyl assignments were not possible due to signal overlap.

To identify the farnesyl resonances, we recorded a ^1^H,^13^C HSQC spectrum on a ^13^C, ^15^N, ^2^H labeled PEX19 sample. However, in these spectra the natural abundance ^13^C correlations of the farnesyl group are obscured by residual correlations from the protein (Fig. S2). According to the glucose specifications used (99% ^13^C, 97% ^2^H) and assuming that all other components of the bacterial growth medium were dissolved in D_2_O, ca. 3% residual ^1^H signal is expected for the protein. On the other hand, the attached farnesyl corresponds to 1% ^1^H signal at natural isotopic abundance. Thus, residual protein and natural abundance farnesyl ^13^C correlations are present at comparable intensities. Therefore, it is difficult to distinguish farnesyl correlations from the residual signals of the protein (Fig. S2).

To circumvent this problem we prepared perdeuterated PEX19 (97% ^2^H) without ^13^C labeling to reduce the occurrence of ^13^C-bound protons in the protein (at natural abundance) to 0.03%. As shown in Fig. 2b, with this labeling scheme the farnesyl resonances are readily detected at natural abundance without interference from protein signals. By comparing the ^1^H, ^13^C correlations of the farnesyl with previous assignments (Umetsu et al. 1999), we could unambiguously identify all chemical shifts. In addition, we recorded a 3D ^13^C-edited NOESY spectrum using non-uniform sampling (Hyberts et al. 2012) and a ^13^C spectral window spanning the allylic methylene and vinyl methyl frequencies. The short-range NOE correlations observed in this experiment further confirmed the farnesyl chemical shift assignments (Fig. 2b). The ^13^C frequencies of the vinyl methyl groups in combination with their NOE correlations established and confirmed the stereochemistry of (2*E*,6*E*)-farnesyl. The methyl groups of the terminal isoprenoid unit were stereospecifically assigned based on their ^13^C chemical shifts. The *trans* methyl group at C14 has an upfield shifted carbon frequency that is in the same range as methyls C4 and C10 that are also in *trans* (~ 20 p.p.m.), and the *cis* methyl group (C15) has a downfield shifted carbon frequency (~ 28 p.p.m.). In support of the stereochemistry and our stereospecific assignments only the *cis* methyl group (C15) gives an NOE to the olefinic proton of the same isoprenoid unit (Fig. 2b). This correlation is not present for any of the *trans* methyl groups (C4, C10, and C14).

Finally, we compared the farnesyl fingerprint spectrum when covalently linked to PEX19 with that of the precursor, farnesyl pyrophosphate in methanol solution. Surprisingly, the chemical shifts are very similar (Fig. 2b). The only differences involve the allylic and olefinic atoms of the first isoprenoid unit and reflect the local microenvironment induced by the neighboring phosphates in pyrophosphate or the protein. The large carbon difference of the first allylic group is due to the different heteroatom attached to when in pyrophosphate or in cysteine. The other chemical shifts are highly comparable, suggesting that the farnesyl moiety experiences a protein hydrophobic environment similar to the organic solvent (Fig. 2b).

### Protein-farnesyl NOE correlations

To obtain protein-farnesyl NOEs we initially recorded isotope-filtered and -edited NOESY experiments with a sample of PEX19 uniformly labeled with ^15^N and ^13^C. These experiments indicated a large number of NOE contacts between protein methyl groups and farnesyl. However, the analysis was complicated by limited spectral resolution in the ^13^C dimension and severe overlap for some of the protein methyl frequencies. PEX19 contains a large number of aliphatic amino acids, including 17 leucine, 9 methionine, and 6 isoleucine residues, for which we could not obtain complete and unambiguous chemical shift assignments using conventional NMR methods and inspection of NOESY spectra (Fig. S3).

**Fig. 3:**
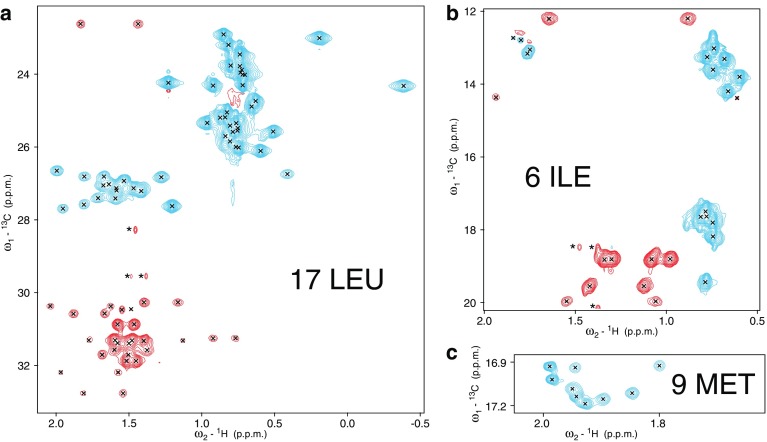
NMR spectra recorded for amino acid selectively-labeled, perdeuterated and uniformly ^15^N-labeled PEX19 protein with unlabeled farnesyl covalently attached. **a** Constant time ^13^C,^1^H HSQC spectrum of perdeuterated PEX19 expressed with ^1^H/^15^N/^13^C leucine. **b** Constant time ^13^C,^1^H HSQC spectrum of perdeuterated PEX19 expressed with ^1^H/^15^N/^13^C isoleucine. **c**^13^C,^1^H HSQC spectrum of perdeuterated PEX19 expressed with ^1^H/^13^Cε methionine. In all cases the Cα proton of the amino acid precursor has been replaced by a deuteron of the solvent. All ^1^H–^13^C resonance pairs are observed. Positive peaks are coloured cyan and negative peaks are coloured red. Asterisks indicate natural abundance methyl crosspeaks of the attached farnesyl

To complete chemical shift assignments and resolve the remaining ambiguities we prepared perdeuterated PEX19 with protonation of specific amino acid side chains or methyl groups (Metzler et al. 1996; Tugarinov et al. 2004). For leucine and isoleucine uniformly ^15^N/^13^C labeled amino acid was used, whereas for methionine a precursor with specific ^13^C labeling of the ε carbon was employed. As shown in Fig. 3, amino acid selective labeling reduces spectral overlap and enabled unambiguous analysis of each residue. Two important aspects of the amino acid selective labeling employed is that no ^13^C scrambling occurs for Leu, Ile, and Met as they are end products of their metabolic pathway (Lacabanne et al. 2017). Also, Hα protons are replaced by deuterons during protein synthesis, as reported previously (Metzler et al. 1996; Crespi et al. 1968; Crespi and Katz 1969; LeMaster 1989) (Fig. S4).

We compared the intensities of Cα–Hα and Cγ–Hγ correlations in a ^1^H, ^13^C HSQC spectrum of the leucine-labeled sample and estimated the level of deuteration at the α position to ~ 97%, consistent with the deuterium enrichment in the growth medium. In ^13^C-edited 3D NOESY experiments all carbon frequencies are resolved, especially those of the methyl groups (Fig. 3). Although no Hα correlations are observable, we were able to assign all leucine ^1^H and ^13^C signals and confirm the isoleucine and methionine assignments based on intraresidue NOEs.

**Fig. 4:**
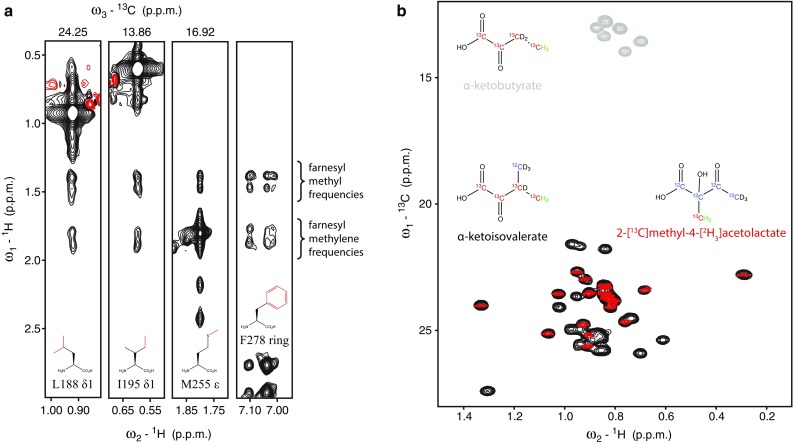
Labeling schemes for mapping protein-farnesyl contacts. **a** Strips from ^13^C-edited NOESY spectra recorded on samples specifically labeled for the methyl groups of leucine, isoleucine, and methionine (highlighted in red in the schematic representation of amino acids) and the aromatic portion of a 2D homonuclear NOESY spectrum on a PEX19 sample reversed labeled for phenylalanines. **b**^13^C, ^1^H HSQC spectra recorded on ILV (U-[^2^H,^12^C]; Ileδ^1^-[^13^CH_3_] gray; Leu/Val-[^13^CH_3_] black) and on (U-[^2^H,^12^C]; Leu/Val-[^13^CH_3_]^pro−S^ red) PEX19 samples

A large number of NOEs are observed between methionine residues and olefinic as well as vinyl methyl groups of the farnesyl moiety. Allylic methylene proton signals overlap with the diagonal peaks of the methionine methyl group (Fig. 4a), and thus NOEs could not be assigned. In some favorable cases farnesyl NOEs from leucine and isoleucine spectra were resolved as well, but most cross peaks are masked by the stronger intraresidue NOE correlations.

To obtain NOE correlations to aromatic protein signals we prepared a perdeuterated protein sample containing protonated phenylalanine residues (Vuister et al. 1994). The aromatic proton frequencies of the four phenylalanines are well resolved from the farnesyl ones, and a 2D homonuclear NOESY spectrum recorded in D_2_O yielded NOEs to farnesyl from one of the aromatic rings (Fig. 4a). Since there is no degradation pathway for Phe in *E. coli*, no scrambling was observed for the aromatic sidechain (Lacabanne et al. 2017), but similarly to Leu and Ile the α protons were replaced by deuterons of the medium during bacterial protein production.

Using this strategy complete and unambiguous methyl assignments were obtained for PEX19. We next prepared an ILV-labeled PEX19 sample by adding α-ketobutyrate and α-ketoisovalerate in the growth media for selective methyl protonation of valine, leucine and δ1 group of isoleucine (Tugarinov et al. 2004). As shown in Fig. 4, these protein frequencies are distinct from the farnesyl ones. Therefore, a large number of protein-farnesyl NOE correlations could be unambiguously assigned, which would otherwise be masked by protein–protein correlations. Finally, an acetolactate precursor was used to obtain stereospecific assignments of methyl groups for leucine and valine residues (Gans et al. 2010) (Fig. 4b).

### Structure calculation

Chemical shift assignments for the 15.5 kDa farnesylated PEX19 protein were complete to 99%. All protein heteronuclear spin pairs were resolved in NOESY spectra, and for Leu and Ile residues in spectra of selectively labeled samples described above. The proton resonances of the farnesyl group were correctly identified, but their chemical shift degeneracy, in particular between equivalent positions in the second and third isoprenoid units, resulted in ambiguity as to which farnesyl atoms of the second and third isoprenoid units are involved in a given NOE. Only the frequencies of the first isoprenoid unit (C1, C2, and C4) and the *cis* methyl group of the third isoprenoid unit (C15) could be resolved (for nomenclature see Fig. 2) in the 3D NOESY spectra. NMR signals corresponding to these farnesyl atoms were manually assigned in all spectra. Signals intensities of the NOE correlation cross peaks were automatically calibrated and converted to distance restraints with an optimized average-distance-parameter of CYANA (Guntert 2009). In a first round of structure calculations, NOE assignments of cross-peaks involving these farnesyl atoms were fixed and the remaining farnesyl NOEs were treated as ambiguous. By reference to the preliminary three-dimensional structure we could uniquely assign cross-peaks to the methyl (C10 and C14) and olefinic atoms (C7 and C12) of the second and third isoprenoid unit that have identical proton chemical shifts. In an iterative procedure additional peaks were held constant to account for distance restraints involving these farnesyl atoms. The NOE correlations to the methylene atoms of the second and third isoprenoid unit (C5, C6, C9, and C11) were assigned last in an automated fashion by using a stringent chemical shift tolerance of 0.015 ppm. Only two of these peaks were excluded by the program due to large violations of the corresponding distance restraints in the resulting structures. Both unassigned peaks matched methylene frequencies but they were singular and just above the noise level. On the contrary, all methylene correlations assigned by the program were present as pairs and corresponded to sequential methylene atoms (C5 and C6 or C9 and C11) (Fig. 4a).

**Fig. 5:**
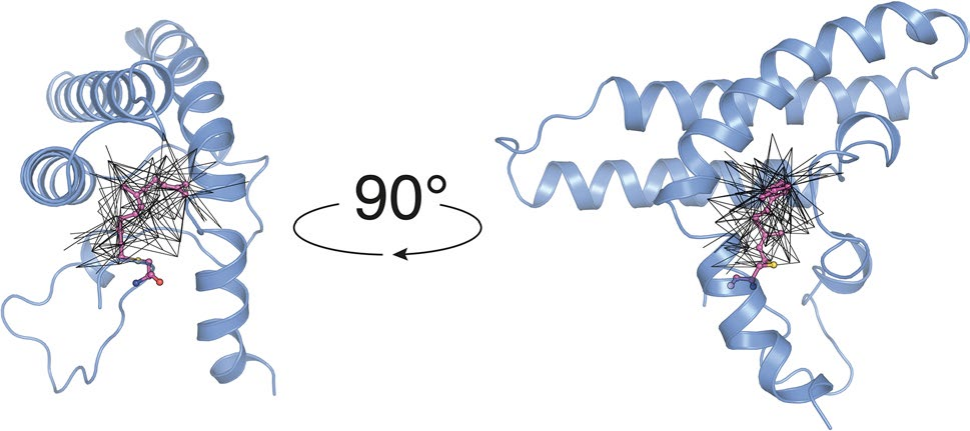
Two views of the solution NMR structure of farnesylated PEX19 (PDB:5LNF). Each black line corresponds to an NOE distance restrain between the protein and the farnesyl moiety. The distance network comprises 203 NOEs recorded on samples using various labeling schemes

Table 1 lists the peak assignment completeness for data recorded in order to map selectively the PEX19-farnesyl contacts. None of the original NOE cross peaks that were picked above a certain threshold were pruned during the iterative assignment strategy. By using a step-by-step semi-automated approach, we were able to collect 203 distance restraints between PEX19 and farnesyl, amounting on average to 17 distance restraints per farnesyl atom. The obtained NOEs are highlighted onto the final structure in Fig. 5.

**Table 1:** Protein samples for collecting NOEs to farnesyl

Precursor (with perdeuterated PEX19 and unlabeled farnesyl)	NOESY spectra^a^	Peaks^b^	Assigned (%)
^1^H/^15^N/^13^C leucine^c^	^13^C-edited 3D NOESY	390	384 (98%)
^1^H/^15^N/^13^C isoleucine^c^	^13^C-edited 3D NOESY	129	127 (98%)
^1^H/^13^Cε methionine	^13^C-edited 3D NOESY	34	34 (100%)
Phenylalanine	2D NOESY	26	26 (100%)
3-[^2^H_3_]methyl-3-[^2^H]-1,2,3,4-[^13^C]-α-ketoisovalerate and 3-[^2^H_2_]-1,2,3,4-[^13^C]-α-ketobutyrate	^13^C-edited 3D NOESY	96	94 (98%)
2-[^13^C] methyl-4-[^2^H_3_]-acetolactate^d^	–	–	–

^a^Leucine, isoleucine and a-ketoacids were recorded in a constant-time fashion

^b^Each set of peaks contains protein–protein NOEs in addition to protein-farnesyl NOEs

^c^Unassigned peaks involve NOEs to protein amide protons

^d^Due to low sample concentration 3D NOESY spectra were not feasible

These data were sufficient for determining a high-resolution structure that revealed a bent conformation of the lipid moiety buried inside a hydrophobic cavity of the protein (PDB:5LNF) (Emmanouilidis et al. 2017).

## Conclusion

We report an optimized protocol for NMR structure calculation of proteins, where ligands or covalently attached modifications cannot be readily obtained in isotope-labeled form. The approach combines specific ^13^C, ^15^N and ^2^H isotope labeling with tailored NMR experiments for chemical shift and NOE assignments to overcome problems with signal overlap and protein–ligand NOE ambiguities. The approach is generally applicable to other challenging systems, where chemical shift degeneracy and signal overlap render analysis of NOESY spectra difficult and prohibit convergence of structure calculations. Of course, the overall success depends on additional factors unique to the system under investigation, e.g. strength of the interaction, exchange processes induced by binding, number of ligand chemical shifts and their degeneracy. However, the basic principles presented here should enable to assess the complexity of the system, adapt the individual steps accordingly and supplement the workflow with additional experiments, if required.

## Methods

### Protein expression and isotope labeling

PEX19 was expressed from a pETM-11 vector containing an N-terminus His-6 tag followed by a TEV cleavage site. *Escherichia coli* BL21 (DE3) cells were grown at 37 °C in perdeuterated medium supplied with U-[^2^H]-d-glucose (2 g l^−1^) and ^15^NH_4_Cl (1 g l^−1^) as the sole carbon and nitrogen sources, respectively. When OD_600_ reached ~ 0.5, 50 mg of the respective labeled amino acid or 150 mg of the respective precursor was added. Cultures were kept at 37 °C for another hour, then incubated at 20 °C for 15 min and induced with 0.5 mM IPTG. After overnight expression at 20 °C, cells were harvested by centrifugation at 5000 r.p.m., washed with PBS buffer and stored at − 80 °C.

### Protein purification

Cell pellets were resuspended in 30 ml lysis buffer containing 50 mM HEPES (pH 7.5), 300 mM NaCl, 10 mM imidazole, 1 mM tris-(2-carboxyethyl)-phosphine (TCEP), and protease inhibitor mix, and lysed by sonication. After centrifugation at 14,000 g the supernatant was loaded onto Ni–NTA gravity-flow resin (Qiagen) and proteins were eluted with increasing concentrations of imidazole. TEV protease treatment was performed overnight (approximately 250 µg TEV for 10 mg of PEX19) and the His-6 tag was removed by Ni–NTA affinity.

### In vitro farnesylation

Untagged PEX19 was mixed with farnesyl transferase fused to His-6 tag and farnesyl pyrophosphate (Sigma Aldrich) in a buffer containing the appropriate amounts of Zn^2+^ and Mg^2+^ ions (Caplan et al. 1992). The reaction mixture was incubated at 37 °C for 1 h and passed through Ni–NTA to remove the farnesyl transferase. Farnesylated PEX19 was further purified by size exclusion chromatography and stored in NMR buffer (20 mM potassium phosphate, pH 6.5 and 50 mM NaCl). Farnesylation was confirmed by SDS gel electrophoresis, ^1^H,^15^N HSQC NMR spectra, and mass spectrometry (Emmanouilidis et al. 2017).

### NMR spectroscopy

NMR spectra were acquired at 298 K on an Avance 900 instrument equipped with a TCI cryo-probe. Spectra were processed using NMRPipe (Delaglio et al. 1995) and analyzed with Sparky (Goddard and Kneller 1996).

## Electronic supplementary material

Below is the link to the electronic supplementary material.

Supplementary material 1 (PDF 3119 KB)
