# Supplementary material for: Selective isotope labeling for NMR structure determination of proteins in complex with unlabeled ligands
Source: J Biomol NMR. 2019 Apr 30;73(3):183–9. doi: 10.1007/s10858-019-00241-9 (PMC6525670; doi:10.1007/s10858-019-00241-9)
Supplement: Supplementary file 1 — Supplementary material 1 (PDF 3119 KB) [file 10858_2019_241_MOESM1_ESM.pdf]

Supplementary Figure 1

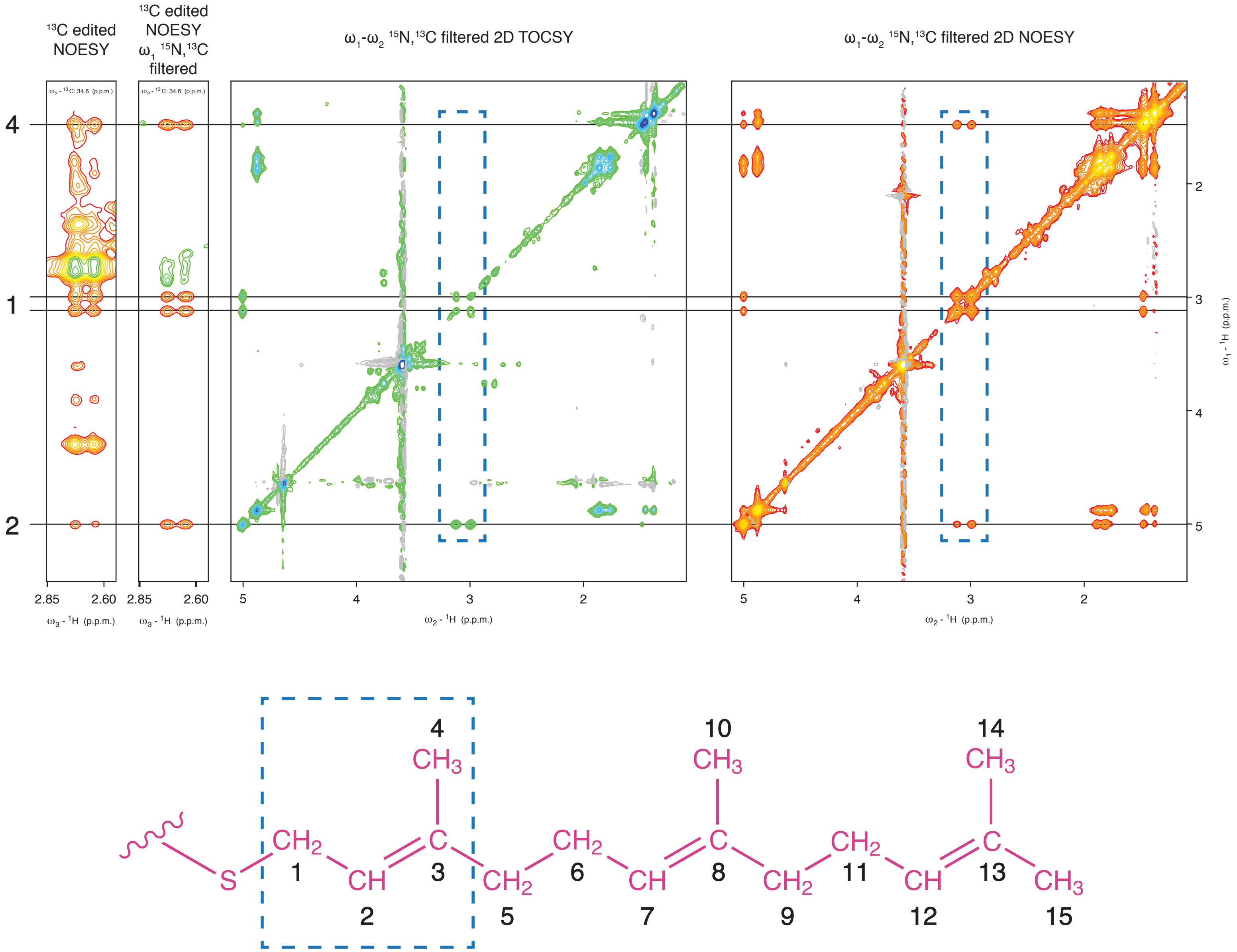

Fig. S1 Proton frequencies of farnesyl when covalently attached to ^{13}C,^{15}N labeled PEX19. Strips on the left show through-space correlations for the methylene protons of the farnesylated cysteine in ^{13}C-edited 3D-NOESY spectra with or without isotope filters in ω_1 (mixing times 70 ms). Middle and right panels show isotope-filtered experiments for detecting through-bond and through-space correlations for the farnesyl moeity, respectively (mixing times 100 ms). Dashed boxes indicate the correlations for the characteristic pair of allylic methylene protons. Horizontal lines mark the resonances of the first isoprenoid unit and numbered according to the farnesyl depiction at the bottom.

Supplementary Figure 2

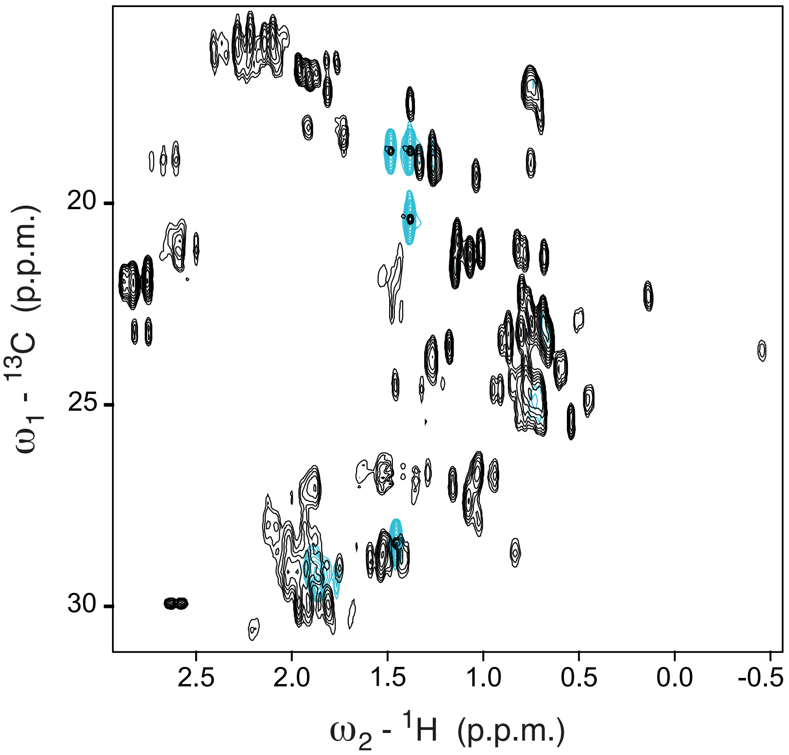

Fig. S2 ^{1}H, ^{13}C HSQC spectra of farnesyl pyrophosphate (cyan) and farnesylated U-[^{2}H,^{15}N,^{13}C] PEX19 (black). Background signals from the protein due to the residual glucose protonation overlap with the natural abundance signals of the farnesyl moiety.

Supplementary Figure 3

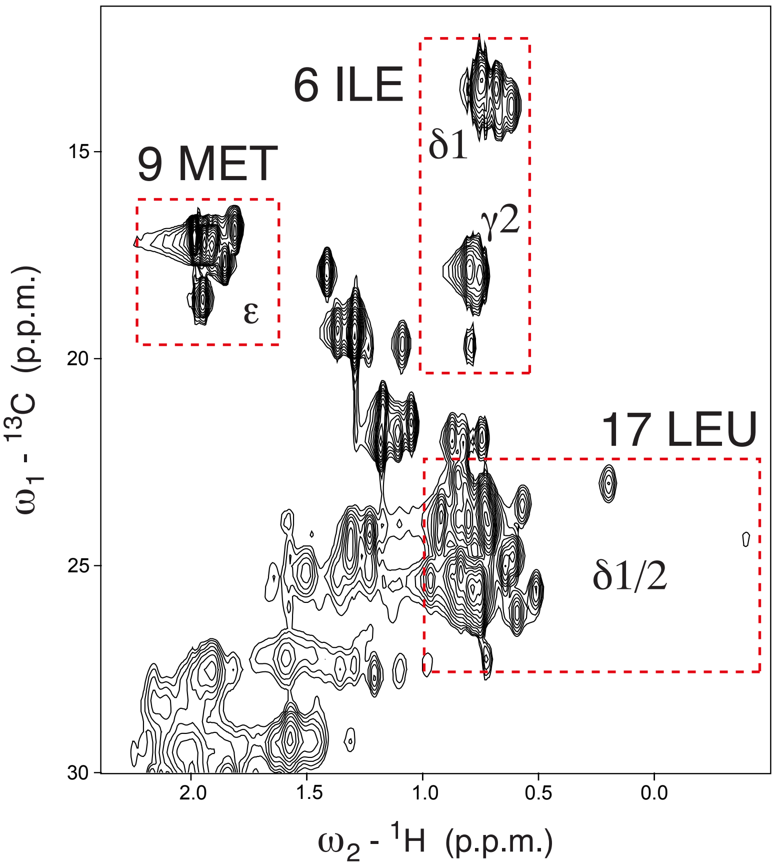

Fig. S3 Methyl region of a ^{1}H, ^{13}C HSQC spectrum of uniformly ^{13}C labeled farnesylated PEX19. Methyl signals of MET, ILE, and LEU are marked (boxed regions). The substantial overlap was resolved using amino acid selective labeling. For comparison see **Fig. 3**.

Supplementary Figure 4

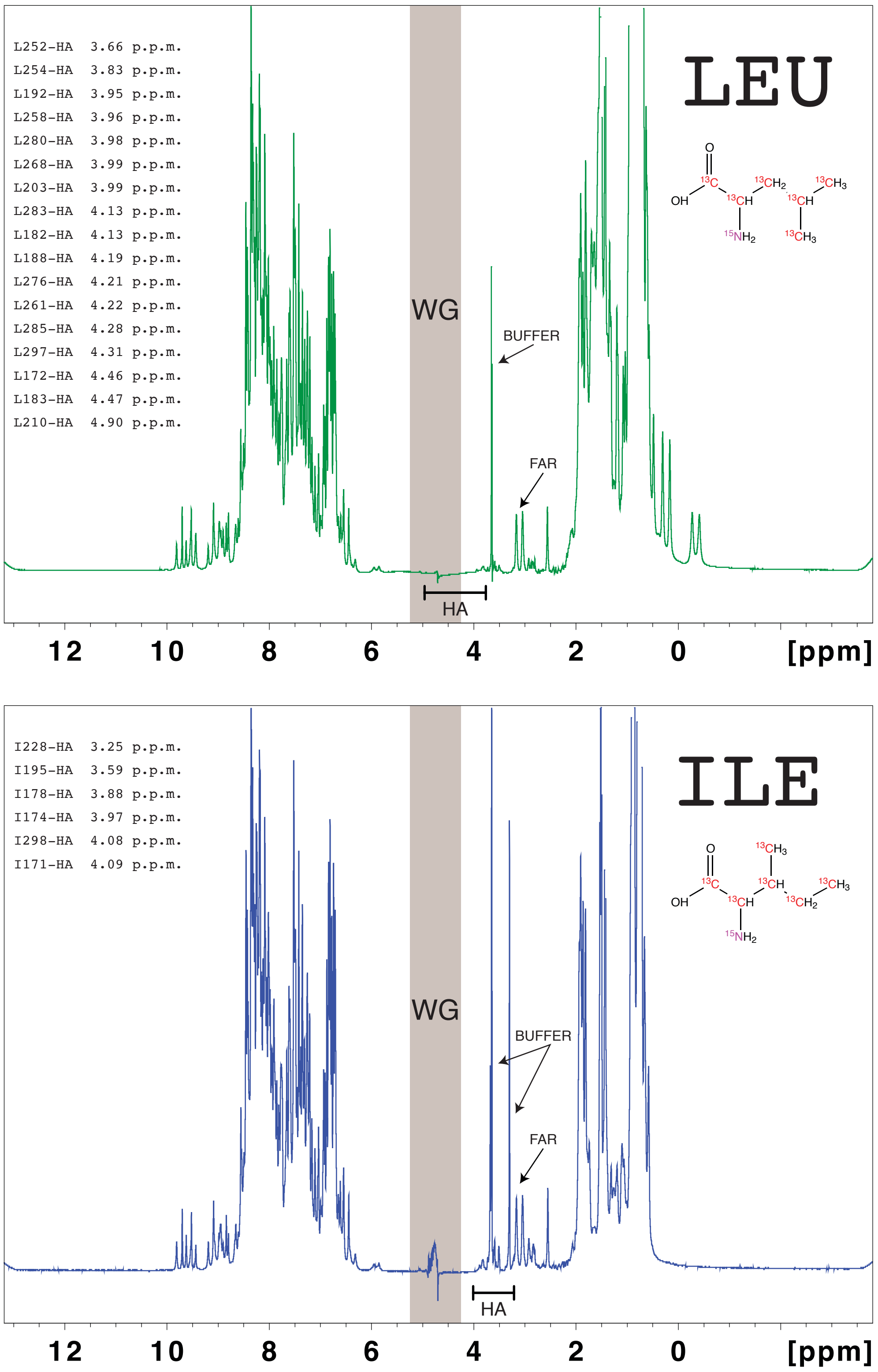

Fig. S4 The α protons of amino acid precursors are replaced by deuterons of the perdeuterated growth media. 1D spectra of perdeuterated farnesylated PEX19 supplemented with $^{1}H/^{15}N/^{13}C$ labeled leucine (top) or $^{1}H/^{15}N/^{13}C$ labeled isoleucine (bottom) during protein expression. In both spectra the α proton frequencies are absent (indicated with a bar at the bottom of each spectrum and their assignments listed in descenting order at left top). Resolved frequencies of farnesyl and buffer are labeled. We note that both spectra were recorded with a watergate pulse sequence that suppresses signals 1 p.p.m. around water (e.g. olefino protons of farnesyl, p.p.m. values 4.8 and 5.1). However the upfield α protons should not be affected by the watergate if they were present. In addition we did not observe any NOE correlation to α proton frequencies in ^{13}C-edited spectra.
